# Trends in Extended-Release and Non–Extended-Release Buprenorphine Dispensing

**DOI:** 10.1001/jamanetworkopen.2025.3158

**Published:** 2025-04-04

**Authors:** Thomas J. Stopka, Netrali Dalvi, Leonard D. Young, Shikhar Shrestha, Danielle DeNufrio Valerio, Alexander Y. Walley

**Affiliations:** 1 Department of Public Health and Community Medicine, Tufts University School of Medicine, Boston, Massachusetts; 2 Prescription Monitoring Program, Massachusetts Department of Public Health, Boston; 3 Boston Medical Center, Boston University Chobanian & Avedisian School of Medicine, Boston, Massachusetts; 4 Bureau of Substance Addiction Services, Massachusetts Department of Public Health, Boston

## Abstract

This cross-sectional study of buprenorphine prescription data in a US state examines relative changes in prescriptions of extended-release and non–extended-release versions, along with prescription characteristics.

## Introduction

Estimated opioid use disorder (OUD) prevalence among Massachusetts adults reached 5.8% in 2020.^1^ Medications for OUD (MOUD) are effective in treating OUD, with buprenorphine and methadone associated with up to 59% reductions in fatal overdose risk.^2^ Weekly and monthly extended-release formulations of MOUD offer additional effective treatment options to OUD patients and providers that may increase the number of patients initiated and retained on MOUD.^3,4^ Since the first extended-release formulation was approved in 2018, the uptake of extended-release buprenorphine in the US has increased.^5^ The goal of this study was to assess extended-release buprenorphine and non–extended-release buprenorphine dispensing trends in Massachusetts from 2018 to 2023.

## Methods

Using Massachusetts Prescription Monitoring Program (PMP) data, we conducted serial cross-sectional analyses to identify all buprenorphine prescriptions between 2018 and 2023, the most recent years for which data were available, by product name and type (tablet, sublingual film, patch, or injectable) and extended-release buprenorphine or non–extended-release buprenorphine formulation.

We assessed patient counts, days’ supply, counts of unique prescribers and pharmacies, and stratified annual trends by age group and gender. We calculated descriptive statistics and Mann-Kendall tests for trends. We followed the Strengthening the Reporting of Observational Studies in Epidemiology (STROBE) reporting guideline for cross-sectional studies. The Tufts Health Sciences institutional review board deemed these analyses not human participants research. *P* < .05 indicated statistical significance in 2-sided tests.

## Results

A total of 114 669 patients were included in analysis (mean [SD] age, 43.3 [11.7] years; 71 640 male [62.5%]). The number of patients receiving any form of buprenorphine decreased between 2018 (55 632 patients) and 2023 (54 897 patients). Patients receiving extended-release buprenorphine increased from 135 to 4742 between 2018 and 2023 (*P* < .001). Concurrently, patients receiving non–extended-release buprenorphine decreased from 54 956 to 51 768 (*P* < .001). Among patients who received extended-release buprenorphine annually, 71.4% to 88.9% had received a prior non–extended-release buprenorphine formulation in the same year, likely during a transmucosal buprenorphine ramp-up period that is recommended prior to initiating extended-release buprenorphine. The number of non–extended-release buprenorphine prescribers increased between 2018 and 2023 (from 3883 to 6512 prescribers) while the number of prescriptions decreased (from 54 956 to 51 768 patients) (Table). Twice as many men received extended-release buprenorphine as women in 2023 (3195 vs 1537, respectively). The ratio was 1.67:1 for non–extended-release buprenorphine in 2023 (32 495 vs 19 421, respectively) (Figure). Age groups 18 to 24 and 25 to 34 years experienced steep annual declines in non–extended-release buprenorphine treatment. All extended-release buprenorphine measures increased annually across all gender and age categories between 2018 and 2023.

**Table.** Prescription Monitoring Program Buprenorphine Statistics

Characteristics	2018	2019	2020	2021	2022	2023	All years
**Extended-release buprenorphine**							
Patients (unique)	135	883	1685	2370	3403	4742	8219
Days’ supply (total)	11 782	101 029	216 759	337 985	477 657	645 350	1 790 562
Pharmacies (unique)	12	18	26	39	41	62	78
Prescribers (unique)	55	180	282	377	471	672	977
Received prior non–extended-release buprenorphine, No. (%)	135 (88.9)	733 (83.0)	1225 (72.7)	1692 (71.4)	2538 (74.6)	3511 (74.1)	77.5^a^
**Non–extended-release buprenorphine**							
Patients (unique)	54 956	58 228	55 023	53 908	53 853	51 768	107 584
Days’ supply (total)^b^	12 314 896	13 018 536	13 547 669	13 190 529	13 033 167	12 736 032	77 840 829
Pharmacies (unique)	1161	1138	1129	1128	1111	1121	1321
Prescribers (unique)	3883	4896	5461	4809	5149	6512	13 419

^a^ Overlap in the receipt of non–extended-release buprenorphine during the same year as extended-release buprenorphine is likely related to the required ramp-up period of receiving transmucosal buprenorphine during the 7 days preceding extended-release buprenorphine initiation.

^b^ The number of days of buprenorphine prescribed across all prescriptions per year.

**Figure.**
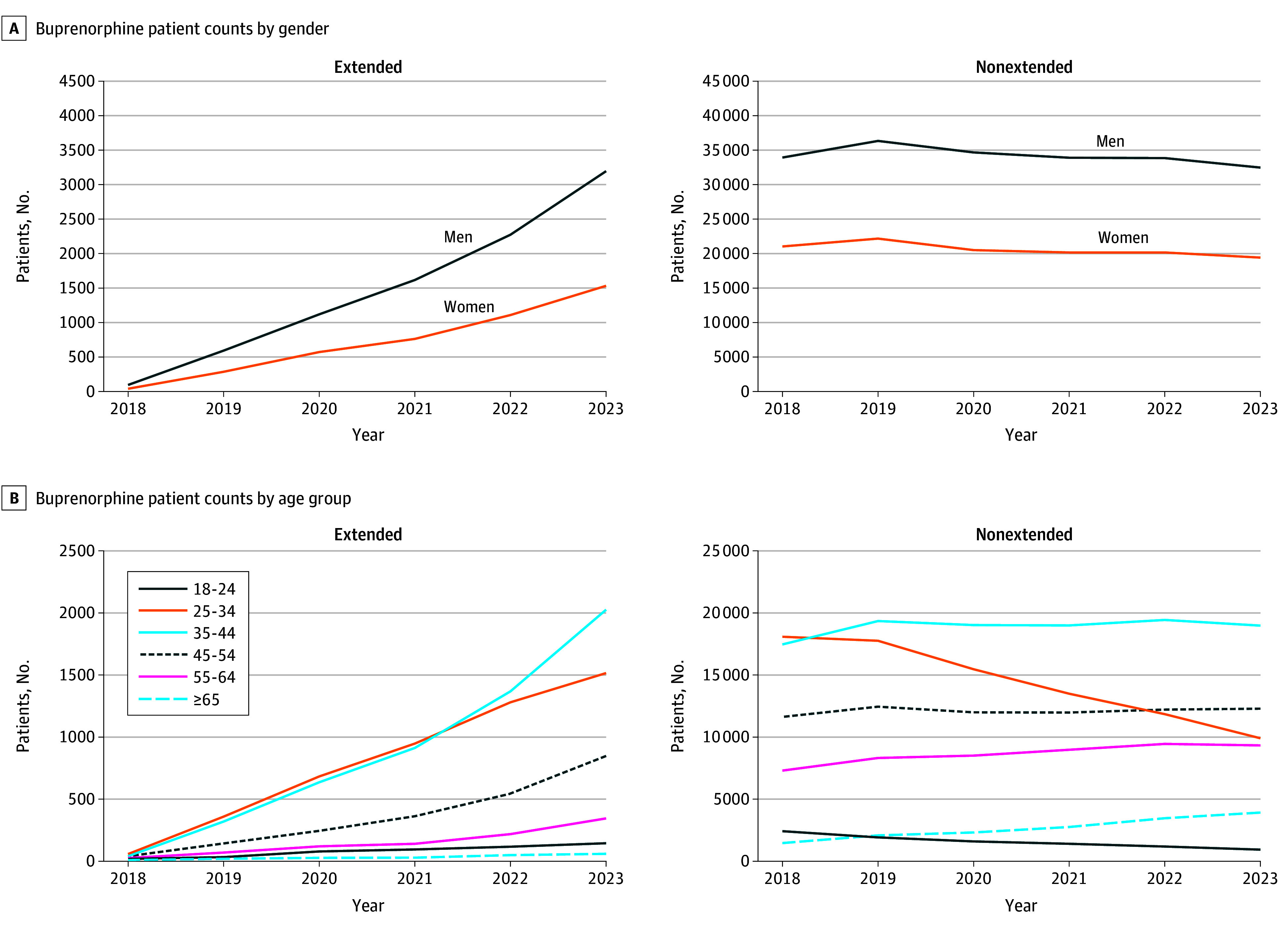
Extended-Release and Non–Extended-Release Buprenorphine Patient Counts by Age and Gender

## Discussion

We observed a steep increase in extended-release buprenorphine patients, days’ supply, pharmacies, and prescribers between 2018 and 2023 and a concurrent decrease in patients receiving non–extended-release buprenorphine. The rise in extended-release buprenorphine patients signals increasing access and a willingness to adopt this treatment option among increasing numbers of people with OUD, but the unanticipated decrease in non–extended-release buprenorphine patients raises concerns. The 2 youngest age strata in the current study experienced a steep decline in non–extended-release buprenorphine treatment, not fully offset by uptake of extended-release buprenorphine. The plateauing and reductions in non–extended-release buprenorphine uptake warrant further study, particularly in younger age groups. Only 25% of people with OUD receive MOUD in the US,^6^ indicating that efforts are needed to improve uptake of buprenorphine and other MOUD.

This study has 2 major strengths: (1) It is among the first to examine extended-release buprenorphine and non–extended-release buprenorphine dispensing trends for an extended period outside a clinical trial; (2) analyzing both extended-release buprenorphine and non–extended-release buprenorphine provided a robust comparator, uncovering unexpected decreases in non–extended-release buprenorphine dispensing. A limitation is that medications ordered and medically billed in clinics are not required to be reported to the PMP. While most community clinics rely on specialty pharmacies, which do report, underreporting of burprenorphine patients and prescriptions is possible. Findings may not be generalizable beyond Massachusetts.

## Conclusions

The steep increase in people treated with extended-release buprenorphine indicates increasing access and acceptability of extended-release formulations among people with OUD in Massachusetts. To reduce opioid overdose deaths and other consequences of OUD, further research on improving access and acceptability of all effective MOUD is needed.

## Notes

Supplement.
Data Sharing Statement
