# Error in Figure

**DOI:** 10.1001/jamanetworkopen.2025.17382

**Published:** 2025-05-23

In the Research Letter titled “Trends in Extended-Release and Non–Extended-Release Buprenorphine Dispensing,”^1^ published April 4, 2025, there was an error in the Figure. In panel A, the lines indicating the numbers of men and women receiving non–extended-release buprenorphine were transposed. This article has been corrected.^1^
