# Supplementary material for: Trends in Extended-Release and Non–Extended-Release Buprenorphine Dispensing
Source: JAMA Netw Open. 2025 Apr 4;8(4):e253158. doi: 10.1001/jamanetworkopen.2025.3158 (PMC11971669; doi:10.1001/jamanetworkopen.2025.3158)
Supplement: Supplement. — Data Sharing Statement [file jamanetwopen-e253158-s001.pdf]

Data Sharing Statement

Stopka. Trends in Extended-Release and Non–Extended-Release Buprenorphine Dispensing. *JAMA Netw Open*. Published April 04, 2025. doi:10.1001/jamanetworkopen.2025.3158

Data
Data available: No

Additional Information
Explanation for why data not available: The data from the Massachusetts Prescription Drug Monitoring Program (PMP) are not publicly available, but may be made available upon request to the PMP.
